# Metabolic stress controls mutant p53 R248Q stability in acute myeloid leukemia cells

**DOI:** 10.1038/s41598-019-42220-y

**Published:** 2019-04-04

**Authors:** Nerea Allende-Vega, Martin Villalba

**Affiliations:** 1 IRMB, Univ Montpellier, INSERM, CHU Montpellier, Montpellier, France; 2 IRMB, CHU Montpellier, Montpellier, France

## Abstract

Eliminating mutant p53 (mt p53) protein could be a useful strategy to treat mt p53 tumors and potentially improve the prognosis of cancer patients. In this study, we unveil different mechanisms that eliminate p53-R248Q, one of the most frequent mutants found in human cancers. We show that the Hsp90 inhibitor 17-AAG eliminates R248Q by stimulating macroautophagy under normal growth conditions. Metabolic stress induced by the pyruvate dehydrogenase kinase-1 (PDK1) inhibitor dichloroacetate (DCA) inhibits the macroautophagy pathway. This induces the accumulation of R248Q, which in addition further inhibits macroautophagy. Combination of DCA and 17-AAG further decreases the autophagy flux compared to DCA alone. Despite this, this co-treatment strongly decreases R248Q levels. In this situation of metabolic stress, 17-AAG induces the binding of p53-R248Q to Hsc70 and the activation of Chaperone-Mediated Autophagy (CMA), leading to higher R248Q degradation than in non-stress conditions. Thus, different metabolic contexts induce diverse autophagy mechanisms that degrade p53-R248Q, and under metabolic stress, its degradation is CMA-mediated. Hence, we present different strategies to eliminate this mutant and provide new evidence of the crosstalk between macroautophagy and CMA and their potential use to target mutant p53.

## Introduction

Wild-type p53 (wt p53) was refereed once as “the guardian of the genome” for its important role as a tumor suppressor gene^1^. Today p53 is not only known as a tumor suppressor but also a master regulator of many cellular processes such as cell cycle, apoptosis, DNA repair, inflammation and metabolism^2^. The *p53* gene is the most frequent target for mutation in human cancer, including hematological malignancies^3^. The frequency of *p53* mutations in acute myeloid leukemia (AML) is approximately 10%. However, in AML with complex karyotype, the rate of *p53* mutations and/or deletions is almost 70%^4^. Furthermore, *p53* mutations are associated with poor prognosis and decreased survival in AML.

Mutations are found in all coding exons of the *p53* gene, but most of them are located in the DNA-binding domain, with the most common in codons 175, 245, 248, 273 and 282. These are the “hot spot” residues, which are very frequently mutated in all types of cancer^5^. These mutations do not always correlate with loss of function of p53 and can actively promote tumor growth by gain-of-function (GOF) mechanism^6–8^. The important role of GOF by mutant p53 (mt p53) is further supported by the finding that patients carrying missense mutation and expressing mt p53 in the germline have a significantly earlier cancer onset than patients with mutations in *TP53* that result in loss of p53 protein^9,10^. Moreover, mt p53 accumulation is critical for p53 oncogenic GOF that actively contributes to cancer development and progression^11^.

R248 is mutated into three amino acids R248Q, R248W and R248L^12^. Interestingly, p53-R248Q, but not p53-R248W, confers invasive ability when overexpressed in p53-null cells^13^. Thus, not only the position of the mutation but also the nature of the substitution may influence the activity of the resulting mt p53 protein. In fact, mutant R248Q induces more aggressive tumors in mice compare with other hotspot mutants^14–16^. R248Q has a greater tendency to aggregate and can seed the aggregation of wt p53. In breast cancer samples, R248Q aggregates into prion-like amyloid oligomers sequestrating and inactivating wt p53^17^. Codon 248 of the p53 protein is most frequently mutated in pancreatic tumors (based on cBioPortal), in lymphomas^18^, myelodysplastic syndromes (MSD) and AML^19,20^. In summary, it is essential to further study mechanisms reducing the function of this p53 mutant, but with a minimal effect on wt p53.

Wt p53 stability is mainly control by the proteasome-ubiquitin pathway, however it is still unclear which pathway degrades mt p53. In response to different stresses, both wt and mt p53 accumulate in cells. While wt p53 returns to basal level following recovery from stress, mt p53 remains stable^21^. Certain mt p53 proteins accumulate to high levels in tumor cells^22^ due to its interaction with the chaperones Hsp70 and Hsp90. Hsp90 inactivates the E3 ligases MDM2 and CHIP, impairing proteasomal degradation of mt p53^23^. mt p53 degradation also occurs by different types of autophagy: macroautophagy and Chaperone-Mediated Autophagy (CMA)^24^. Macroautophagy, induced by glucose restriction or by proteasomal inhibition, promotes mt p53 degradation^25^. When nutritional deprivation inhibits macroautophagy, CMA is activated and induces mt p53 degradation^26^. For further complexity, mt p53 can inhibit autophagy^27,28^.

One approach to target mt p53 is to reduce mt p53 levels with little effect on wt p53 using compounds that promote degradation of mt p53 such as the Hsp90 inhibitor 17-AAG^23,29^. 17-AAG is a geldanamycin analogue, currently in clinical trials as anticancer drug that triggers the activation of a heat shock response, promotes proteasome degradation and induces the autophagic pathway^30–32^.

In this study, we uncover different mechanisms that promote mutant p53-R248Q depletion in different cellular contexts. In tumors growing in normal, no stress, conditions, 17-AAG eliminates R248Q through macroautophagy. However, in tumors with macroautophagy inhibition and high stability of mt p53, 17-AAG still was able to induce mt p53 degradation through CMA. Also we showed that metabolic stress caused the pyruvate dehydrogenase kinase-1 (PDK1) inhibitor dichloroacetate (DCA) promotes higher accumulation and stabilization of R248Q protein by increasing its interaction with the Hsp90 chaperone machinery. Furthermore, accumulation of R248Q prevents macroautophagy by inhibiting the expression of several macroautophagy genes. Our data demonstrate that there is a negative feedback loop between macroautophagy and mt p53. Under DCA-induced metabolic stress, when macroautophagy is largely reduced, 17-AAG induces mt p53 degradation through CMA.

## Results

### Different effect of DCA and 17-AAG on R248Q stability

DCA causes metabolic stress by inhibiting PDK1 in AML cells^33,34^. This forces cells to decrease glycolysis and increase oxidative phosphorylation (OXPHOS)^33–35^. DCA induces wt p53 transcriptional activity via AMPK and its efficacy to cause cell cycle arrest depends on p53 status^29^. Besides, we observed that both wt p53 and mt p53 protein levels accumulated after DCA treatment including in the NB4 cell line, which carries p53 R248Q^29^. We extensively investigated the functional activity of wt p53 after DCA treatment and found that p53 induced cell cycle arrest in G0/G1 phase, although failed to induce programmed cell death (PCD). Cell cycle arrest involved p53 transcriptional activity because we observed upregulation of *MDM*2 and *p21* mRNAs^29^ (Supplementary Fig. 1a). We also described in this work that DCA-induced metabolic stress depended in wt p53 and involved mRNA expression of its metabolic targets *GLS2*, *SCO2* and *AMBKβ*^29^. Moreover, DCA increased ROS expression and disturbed oxygen consumption^29^.

**Figure 1:**
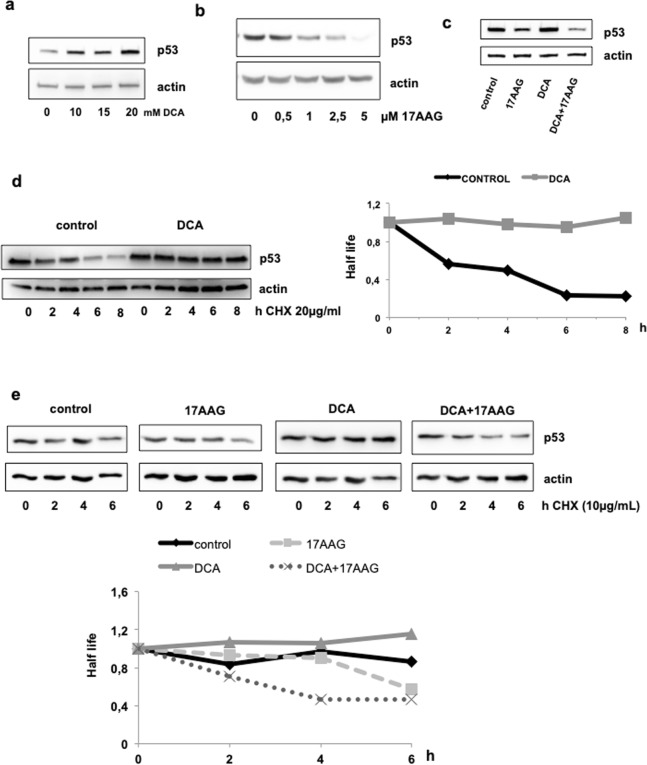
DCA and 17-AAG have different effect on p53-R248Q stability. (**a**) NB4 (mutant p53-R248Q) cells were incubated with the indicated concentrations of DCA for 24 h. p53 protein levels were analyzed by western blot analysis. (**b**) Decrease of p53 protein levels in NB4 cells after treatment with different concentrations of 17-AAG for 24 h. (**c**) NB4 cells were treated with DCA (10 mM) and/or 17-AAG (1 µM) for 24 h before protein analysis by western blotting. (**d**) NB4 cells were treated with 10 mM DCA and after 24 h incubated with 20 μg/mL cycloheximide (CHX) and harvested at the indicated times. At the indicated times after the addition of cycloheximide, the level of p53 was analyzed by western blotting. (**e**) NB4 cells were treated for 24 with 10 mM DCA and/or 17-AAG (1 µM) and after 24 h treated with CHX as in (**d**). Graphs represent the quantification of the western blots.

We confirmed here that DCA induced accumulation of mt p53 protein without affecting *p53* mRNA (Fig. 1a and Supplementary Fig. 1b). Hsp90 inhibition by 17-AAG promoted R248Q degradation (Fig. 1b). Surprisingly, co-treatment with DCA and 17-AAG was more effective than 17-AAG alone (Fig. 1c). This effect was only observed at protein level. *R248Q* mRNA was not affected by DCA + 17-AAG co-treatment (Supplementary Fig. 1c). This shows that DCA effects mainly rely in protein stability as previously proposed^29^.

To understand how these two drugs, with individually opposite effects on mt p53 protein levels, synergy to eliminate R248Q, we study the stability of R248Q in presence of DCA and 17-AAG alone and in combination. First, we determined whether the augmentation in R248Q protein correspond with an increase of stability following DCA treatment. To measure the half-life of mtp53, NB4 cells were treated for 24 h with 10 mM DCA. Next, we added at different times the inhibitor of protein synthesis cycloheximide and analyzed the levels of p53 by western blotting. As shown in Fig. 1d, the stability of R248Q was higher after DCA treatment suggesting that DCA interferes with mt p53 degradation. Then, we examined the stability of R248Q after treatment of DCA and/or 17-AAG. The half-life of R248Q decreased with 17-AAG (Fig. 1e). DCA + 17-AAG co-treatment further reduced R248Q level. These data indicate that decrease of R248Q protein levels is due to decrease in stability upon DCA + 17-AAG treatment and not to inhibition of mtp53 mRNA expression (Supplementary Fig. 1c).

### Hsp90 controls R248Q stability

**Figure 2:**
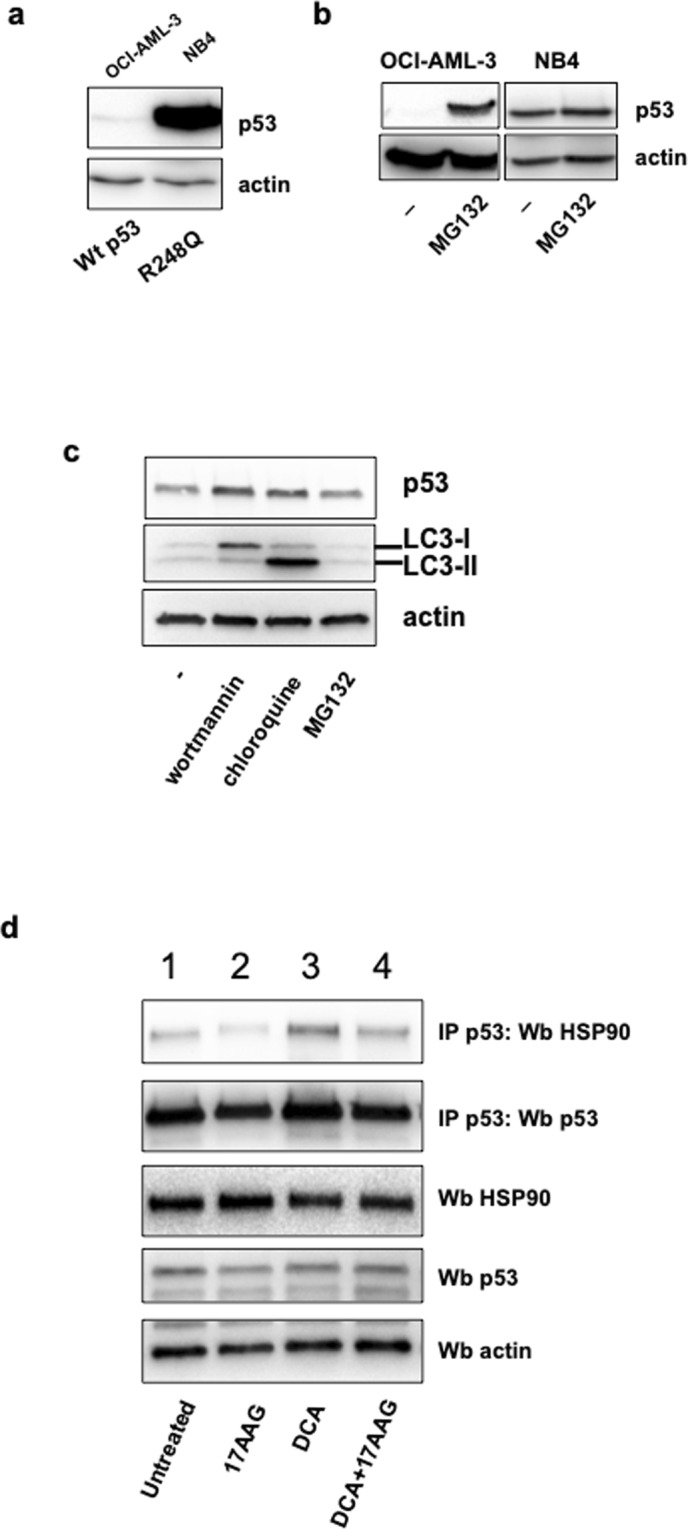
Different mechanisms control wild type and mutant p53 stabilities. (**a**) Comparison of the endogenous p53 protein levels from two AML cell lines with different p53 status, OCI-AML3 cells (wt p53) and NB4 cells (R248Q). (**b**) The proteasome inhibitor MG132 induces accumulation of wt p53 but not of the R248Q mutant protein. NB4 cells were incubated with MG132 (10 µM) for 6 h before analyzing p53 levels. (**c**) Autophagy inhibition induces accumulation of p53-R248Q. NB4 cells were incubated for 4 h with the autophagy inhibitors chloroquine (50 µM) and wortmannin (50 mM) and MG132 before analyzing p53 levels. (**d**) Co-immunoprecipitation of p53 and Hsp90 after treatment with DCA and/or 17-AAG. NB4 cell extracts from cells treated for 8 h with 1 µM 17-AAG and 10 mM DCA were used for immunoprecipitation with the anti-p53 antibody (DO-1) and analyzed by western blotting with anti-Hsp90 or anti-p53 antibodies as indicated.

There are two main routes of protein degradation in eukaryotes, the ubiquitin-proteasome and the autophagy-lysosome pathways. The first predominantly regulates the stability of wt p53. Little is known about the degradation of mutant p53. In general, mt p53 proteins show increased stability compared to the wt protein due to their interaction with the Hsp90 chaperone complex^23^. We compared the endogenous levels of p53 protein from two AML cell lines with different p53 status, OCI-AML3 cells (wt p53) and NB4 cells (R248Q). NB4 cell line showed higher expression of p53 (Fig. 2a). The proteasome inhibitor MG132 induced accumulation of wt p53 but not of p53-R248Q (Fig. 2b). The magnitude of the effect of MG132 treatment indicates that the proteasome is the major route for wt p53 degradation, but not for R248Q. This result suggests that different degradation pathways control the stability of wt p53 and R248Q.

Next, we examined the role of the lysosome pathway by using the lysosome inhibitor chloroquine (CQ) and wortmannin, an inhibitor of the early stage of macroautophagy. Both inhibitors caused accumulation of R248Q protein levels (Fig. 2c). The increase of the macroautophagy markers, LC3-I and LC3-II detected after treatment of wortmannin and CQ respectively, indicates that both compounds were functional. MG132 did not affect LC3-I and LC3-II or mt p53 levels. Hence R248Q degradation is mainly dependent on the lysosomal, but not on the proteasomal pathway.

The molecular chaperone Hsp90 maintains the conformation, stability and activity of several oncogenic proteins including specific mt p53 proteins^36^. As 17-AAG promotes degradation and decreases stability of R248Q (Fig. 1b), we studied the role of Hsp90. We monitored the p53-Hsp90 interaction by co-immunoprecipitation experiments after treatment with DCA and/or 17-AAG (Fig. 2d). This co-immunopecipitation experiment was performed for 8 hours to avoid the complete degradation of p53. NB4 cell extracts were used for immunoprecipitation with the anti-p53 antibody (DO-1) and the eluates blotted against Hsp90. R248Q interacted with the chaperone Hsp90 (Fig. 2d, lane1). As expected, inhibition of Hsp90 with 17-AAG reduced the interaction of R248Q with Hsp90 (Fig. 2d, lane 2) while in contrast DCA increased the R248Q-Hsp90 binding (Fig. 2d, lane 3). DCA + 17-AAG co-treatment decreased Hsp90-R248Q interaction compared to DCA alone (Fig. 2d, lane 4). These results indicate that Hsp90 is an important regulator of R248Q stability.

### 17-AAG induces macroautophagy in AML cell lines

**Figure 3:**
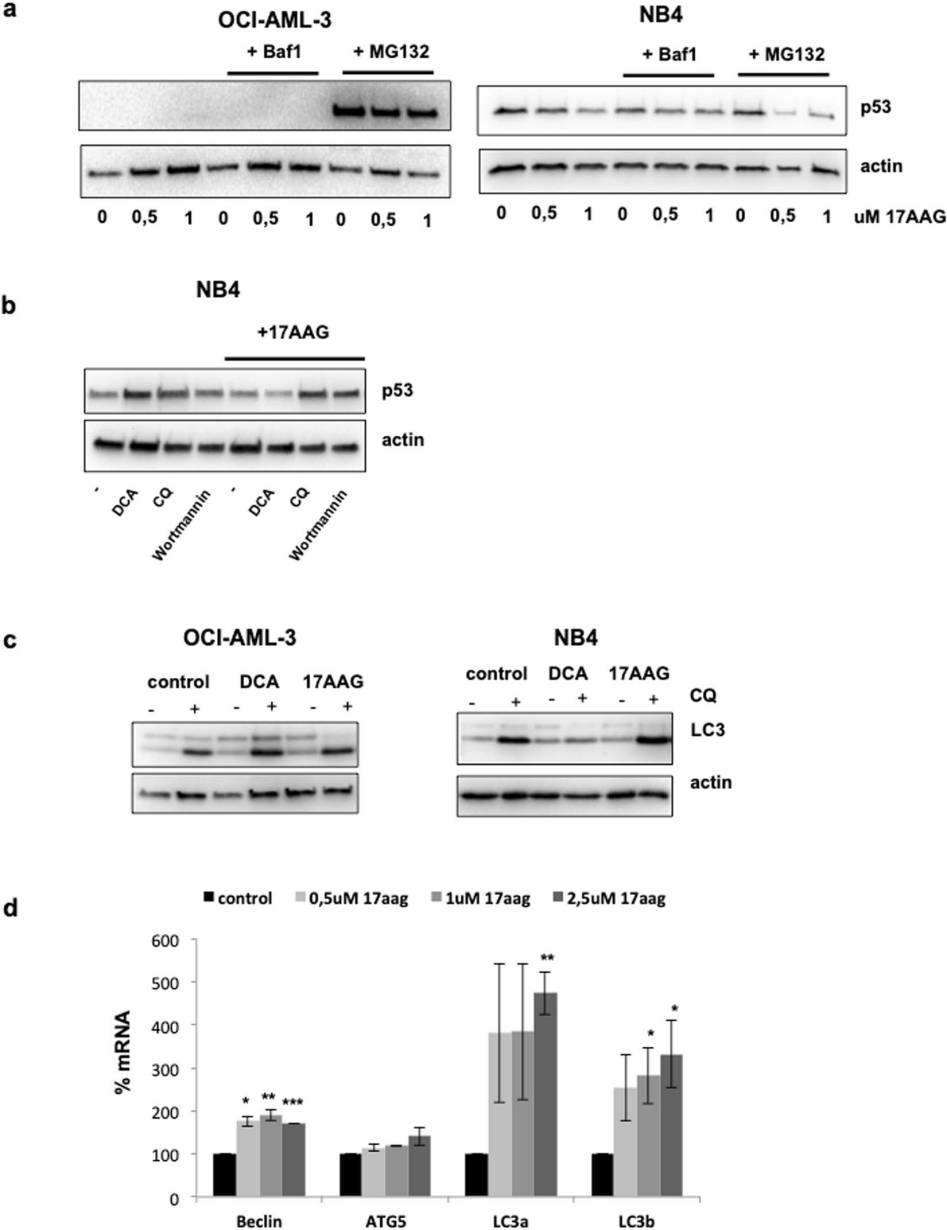
17-AAG induces macroautophagy in NB4 cells. (**a**) OCI-AML-3 (wtp53) and NB4 (R248Q) cells were treated with different doses of 17-AAG for 24 h and then treated with the proteasomal inhibitor MG132 (10 µM) or the macroautophagy inhibitor bafilomycin A1 (Baf A1; 100 nM) for 4 hours before analyzing samples by western blotting. (**b**) NB4 cells were incubated with or without 17-AAG for 24 h and then with two autophagy inhibitors: 50 nM wortmannin and 50 uM chloroquine for 4 h. (**c**) Increase in LC3-II levels after treatment with 17-AAG in NB4 cells. Chloroquine was added for 4 h after treatment for 24 h with 17-AAG (1 µM) or DCA (10 mM). Protein extracts were analyzed by western blotting for the depicted proteins. (**d**) RT-qPCR analysis of autophagy genes (*atg5*, *becn1* and *LC3*) in NB4 cells treated with different concentration of 17-AAG for 24 h; *p < 0.05; **p < 0.01; ***p < 0.001.

17-AAG is currently in clinical trials as an anticancer drug that specifically inhibits Hsp90 functions^32^, but up-regulates other HSPs. 17-AAG promotes mt p53 proteasomal degradation^23^ and autophagy to remove aggregates^31^. To investigate whether 17-AAG enhances proteasomal or lysosomal degradation in wt p53 and mt p53 AML cells, these were exposed to 17-AAG at different doses for 24 h and then treated with the proteasomal inhibitor MG132 or the macroautophagy inhibitor bafilomycin A1 (Baf A1) for 4 hours before analyzing samples by western blotting. Baf A1 is an inhibitor of the late phase of autophagy that prevents the fusion between autophagosomes and lysosomes. In NB4 cells, inhibition of the proteasome with MG132 failed to accumulate mt p53, instead resulted in a reduction (Fig. 3a). In contrast, inhibition of macroautophagy by Baf A1 blocked mt p53 degradation mediated by 17-AAG. In OCI-AML-3 wt p53 was accumulated only in the presence of MG132.

Next, we examined whether the macroautophagy pathway mediates R248Q degradation by 17-AAG. We treated NB4 cells with 17-AAG for 24 h and then with 50 nM wortmannin and 50 uM CQ for 4 h. In contrast to DCA, both wortmannin and CQ were able to rescue degradation caused by 17-AAG (Fig. 3b). This suggests that 17-AAG causes the elimination of R248Q through induction of macroautophagy.

We next determined the effect of 17-AAG and DCA on macroautophagy flux in AML cells with different p53 status. We monitored autophagy flux by the addition of CQ (Fig. 3c). Protein extracts were analyzed by western blotting for LC3-II, a well establish macroautophagy marker. In OCI-AML-3, the autophagy flux was maintained after treatment of any of these drugs. We observed an increase in LC3-II levels after treatment with 17-AAG in NB4 cells (R248Q). Interestingly, a reduction of autophagy flux was observed after DCA treatment (Fig. 3c).

Macroautophagy is a complex sequence of biological events leading to formation, maturation, and fusion of autophagosomes with lysosomes to allow the degradation and recycling of cellular components^37^. All these events are controlled by many autophagy-related genes (ATGs), which are mostly transcriptionally induced by different stimuli such as nutritional deprivation, infections or metabolic and oncogenic stress^38^. To determine the mechanism for the increase in autophagy flux upon 17-AAG treatment in NB4 cells, we monitored mRNA levels of some essential autophagy genes such as *ATG5*, *BECN1* and two different isoforms of *LC3* (Fig. 3d). *BECN1* and *LC3* mRNAs were significantly increased indicating that 17-AAG may up-regulate macroautophagy by increasing the transcription of autophagy genes. Taken together all these results suggest that the degradation of R248Q by 17-AAG is dependent on macroautophagy and not on proteasomal mechanisms. Thus, the stability of wt p53 and mt p53 is controlled by different proteolytic mechanisms.

### DCA inhibits macroautophagy in the presence of R248Q

**Figure 4:**
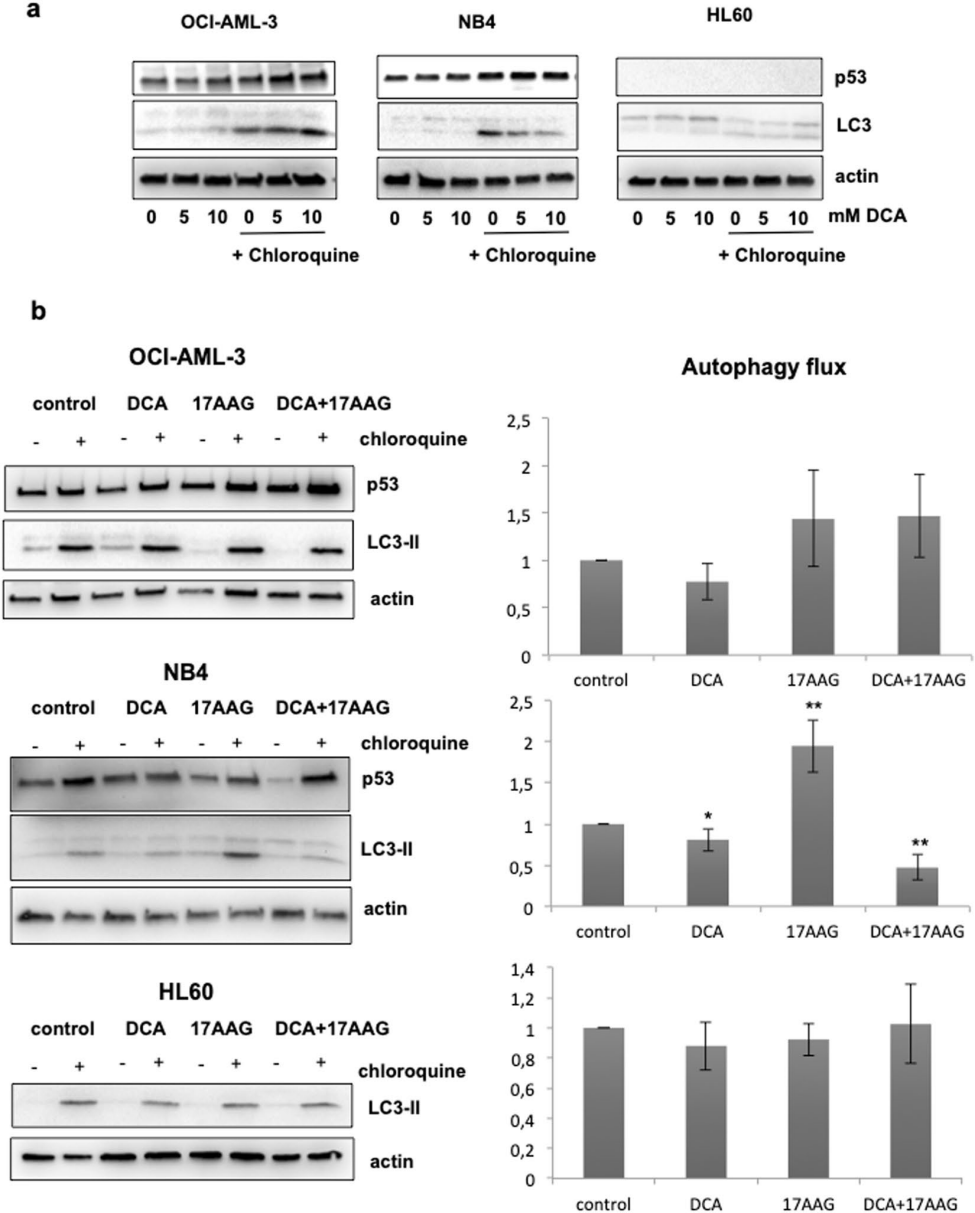
The effect of DCA on macroautophagy depends on the p53 status. (**a**) Autophagy flux after DCA treatment in cell lines with different status of p53. AML cell lines were cultured in the presence of different concentrations of DCA for 24 h followed by 4 h of CQ (50 μM). Autophagy flux was monitored by detection of LC3-II under CQ treatment. (**b**) Effect of 17-AAG alone or in combination with DCA and different status of p53 on autophagy flux. AML cells were treated with 10 mM DCA, and after one hour, 17-AAG was added (1 μM) for 24 h. Autophagy flux was monitored by the addition of 50 μM CQ for 4 h before collecting the cells for western blot analysis. Graphs represent the quantification of the western blots after giving a standard value of 1 to the control.

Depending on the stress and wt p53 location, *i*.*e*. nuclear or cytoplasmic, macroautophagy can be either stimulated or inhibited by this tumor suppressor^39^. Mutant p53, which is mainly cytoplasmic, causes macroautophagy inhibition by repressing the expression of some critical autophagy genes^26–28,40^. We studied the autophagy flux after DCA treatment in cell lines with different status of p53. Autophagy flux was again monitored by detection of LC3-II under CQ treatment. In wt (OCI-AML3) and null (HL60) p53 cell lines, DCA did not basically affect this flux. However, in the NB4 cell line carrying the R248Q p53 mutant, we observed a marked decrease in LC3-II levels (Fig. 4a).

Our results show that the effect of DCA on macroautophagy depends on the p53 status and illustrate that the presence of R248Q may impair macroautophagy. These data also suggest that there is a negative feed back loop between macroautophagy and R248Q. Macroautophagy controls mt p53 protein levels and at the same time mt p53 has an inhibitory effect on macroautophagy.

Next, we monitored the effect of the co-treatment of DCA with 17-AAG on macroautophagy by evaluating the autophagy flux in cells with different p53 status of (Fig. 4b).We did not observe any significant change in autophagy in OCI-AML-3 (wt p53) and HL-60 cells (p53 null) after DCA and/or 17-AAG treatments. Surprisingly, in NB4 cells the combination DCA and 17-AAG further decrease the autophagy flux compared to DCA alone and it was co-related with an enhanced reduction of R248Q protein. Hence, decreasing mt p53 does not rescue the inhibition induced by metabolic stress on macroautophagy flux. Moreover, CQ partially blocked R248Q degradation induced by 17-AAG and DCA + 17-AAG indicating that the lysosomal pathway could be responsible for R248Q removal after these treatments. Therefore, there should be an alternative pathway to degrade mt p53 in conditions where macroautophagy is inhibited.

### Chaperone-mediated autophagy degrades R248Q under metabolic stress

**Figure 5:**
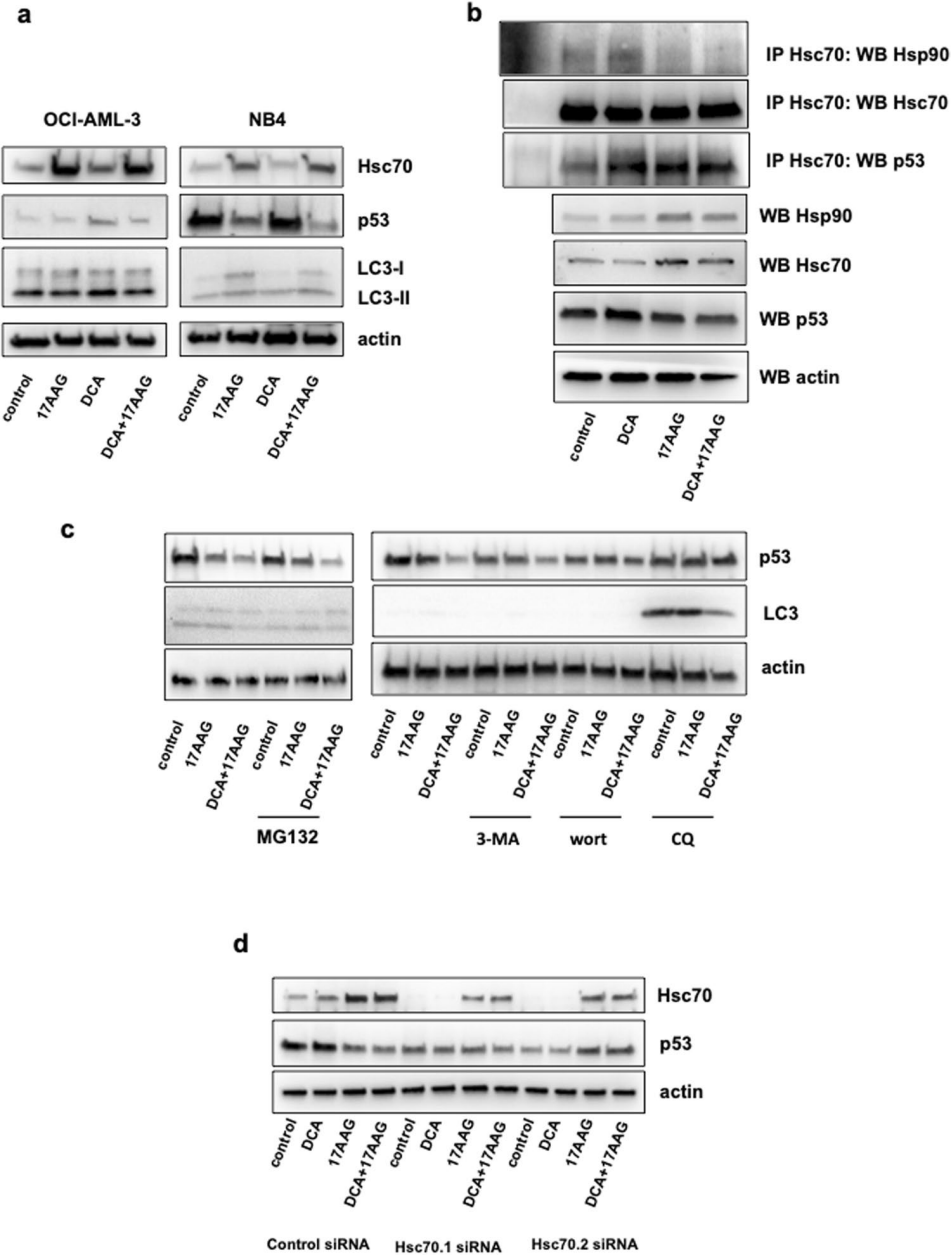
CMA degrades R248Q under metabolic stress. (**a**) Cells were treated with DCA (10 mM) and/or 17-AAG (1 µM) for 24 h before protein analysis by western blotting. (**b**) R248Q interacts with Hsc70 chaperone. NB4 cells were treated with DCA (10 mM) and/or 17-AAG (1 µM) for 8 h, harvested and proteins in the extracts were immunoprecipitated with anti-HSC70 antibody (13D3). Immunoprecipitates were analyzed by Western blotting with anti-Hsc70, anti-p53 and anti-HSsp90 as indicated. (**c**) Only inhibition of autophagy degradation by CQ prevented the elimination of R248Q. NB4 cells were treated with DCA and/or 17-AAG as in (**a**) and with MG132 (10 µM), 3-MA (10 mM), wortmannin (50 nM) and CQ (50 µM) for 12 h. (**d**) Hsc70 is essential for R248Q degradation. NB4 cells were transfected with control siRNA and two siRNA against *Hsc70*. 72 h later they were incubated with 10 mM DCA and/or 17-AAG (1 µM) for 24 h and protein expression was analyzed by western blotting.

CMA can modulate the degradation of multiple Hsp90 client proteins^41^. p53-R248Q significantly decreased with the DCA + 17-AAG combination under conditions where macroautophagy was impaired (Figs 1 and 4b), suggesting a possible role for CMA. In agreement with this hypothesis, 17-AAG increased Hsc70 protein levels even in the presence of DCA in mt and wt p53 cell lines (Fig. 5a). This finding is important because Hsc70 is the only known chaperone to mediate substrate targeting for CMA^42^. To investigate whether CMA is responsible for R248Q degradation, we tested the interaction of R248Q with Hsc70 (Fig. 5b). Both 17-AAG and DCA increased the interaction between R248Q and Hsc70, and this interaction was preserved with the co-treatment DCA + 17-AAG. 17-AAG decreased the interaction of Hsc70 and Hsp90. Our results indicate that inhibiting Hsp90 function has no effect on the binding of R248Q with Hsc70 and neither Hsc70 activity. Importantly, Hsp90 inhibition may be required to induce CMA by 17-AAG. This data also suggests that different pathways are engaged by Hsp90 and Hsc70 to control the stability of R248Q (Fig. 5b).

We further investigated which type of degradation (proteasomal, macroautophagy or CMA) was induced in the presence of DCA and 17-AAG. For this purpose, NB4 cells were treated with DCA and/or 17-AAG with the addition of MG132, 3-Methyladenine (3-MA), wortmannin and CQ (Fig. 5c). We used hence three inhibitors to target autophagy at different steps. 3-MA and wortmannin inhibits autophagosome formation and autophagic sequestration, respectively. CQ blocks lysosomal degradation. Only the presence of CQ prevented the degradation of R248Q, indicating that under these conditions, R248Q degradation is mainly lysosomal and probably through CMA.

Finally, to determine whether the presence Hsc70 is essential for R248Q degradation, we knockdown Hsc70 using two specific siRNAs (Fig. 5d). Downregulation of Hsc70 blocked the degradation of R248Q by 17-AAG and DCA + 17-AAG, indicating that Hsc70 plays a vital role in the stability of R248Q.

Our results propose that CMA could modulate the levels of R248Q under metabolic stress conditions. 17-AAG controls two lysosomal degradation pathways: in normal unstressed conditions, 17-AAG induces macroautophagy and under metabolic stress conditions promotes CMA.

### The inhibitory effect of DCA on autophagy depends on p53 status

**Figure 6:**
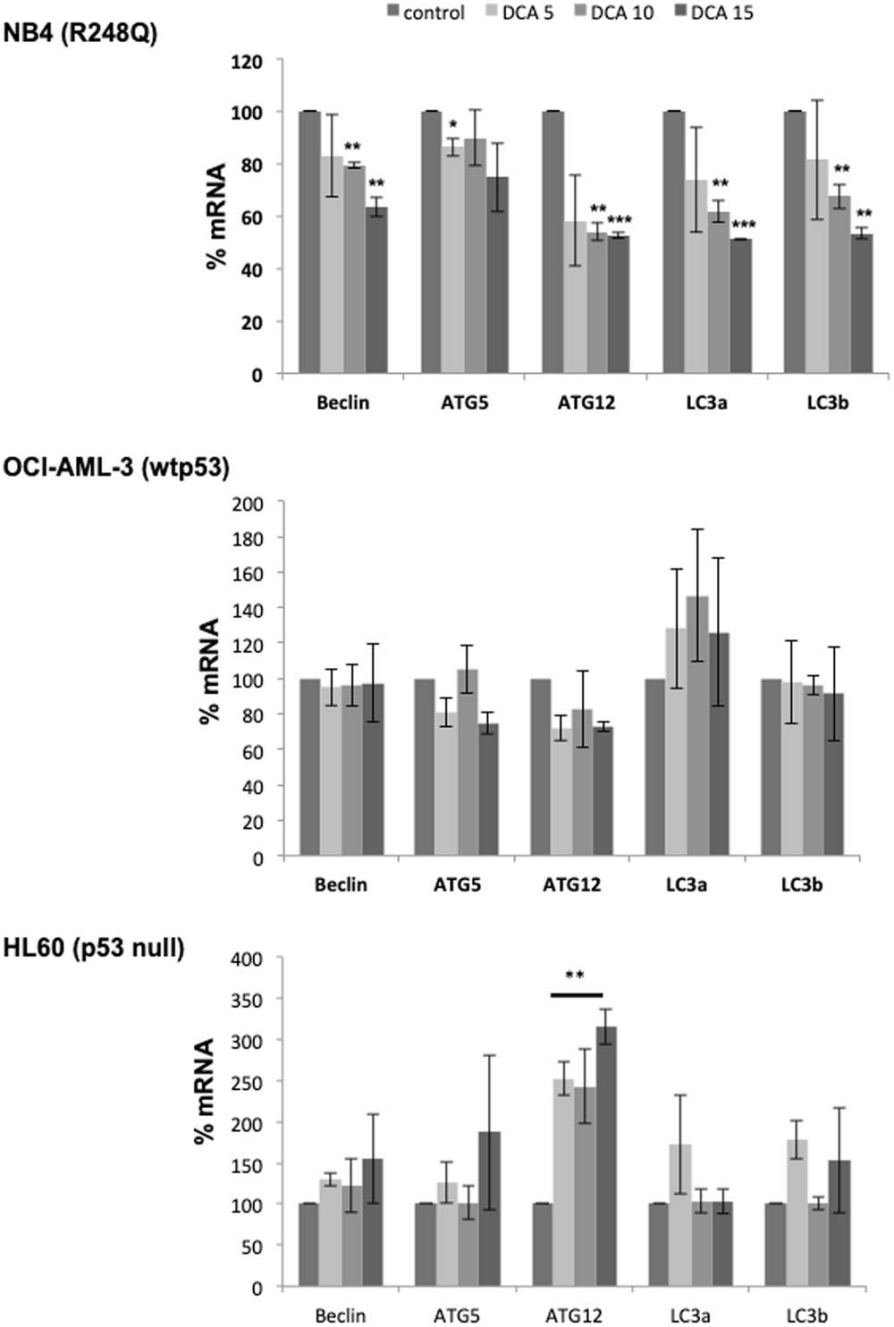
DCA inhibits expression of macroautophagy genes via mutant p53. Different AML cell lines were treated for 24 h with different concentrations of DCA. Expression of autophagy genes (*BECN1*, *ATG5*, *ATG12*, *LC3*) was analyzed by RT-qPCR.

The presence of mt p53 can have an adverse impact on macroautophagy impairing autolysosome formation^27^. Mutants of p53, including R248Q, can counteract autophagy on various phases of the process^28^. To further investigate whether DCA enhances macroautophagy inhibition via mt p53, we monitored the expression of some essential autophagy genes such as *ATG5*, *ATG12*, *BECN1* and two different isoforms for *LC3* (Fig. 6). A decrease of mRNA expression of all these genes was only observed in the NB4 cell line expressing R248Q. No decrease on gene expression was detected in OCI-AML-3 cells (wt p53) or HL60 cell (p53 null). *ATG12* mRNA increased about 2–3 folds in the HL60 cell (p53 null). Hence, DCA inhibits expression of autophagy gene in the presence of R248Q.

### Mutant R248Q has an inhibitory effect on macroautophagy

**Figure 7:**
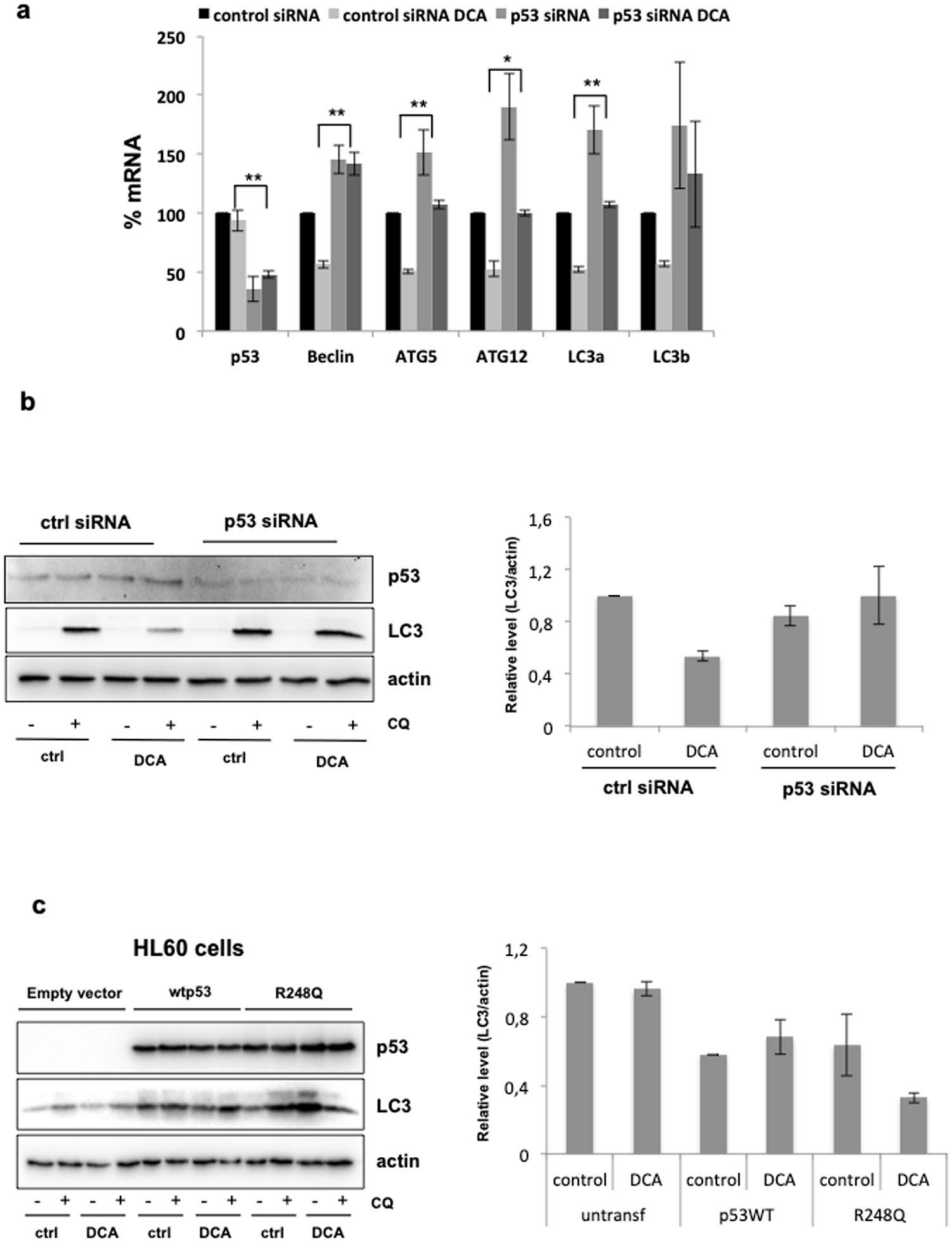
R248Q mediates inhibition of macroautophagy induced by DCA. (**a**) p53 knockdown partially attenuates the inhibitory effect of DCA on the autophagy pathway. NB4 cells were transfected with control siRNA or siRNA targeting p53 and forty hours later were incubated with 10 mM DCA for a further 24 h. The effect on autophagy gene expression was analyzed by RT-qPCR. (**b**) Autophagy flux is restored after R248Q down-regulation. NB4 cells were transfected with control siRNA or p53 siRNA. 72 h later they were incubated with 10 mM DCA for 24 h. Autophagy flux was monitored by detection of LC3-II under CQ treatment. Graphs represent the quantification of the western blots of 3 experiments. (**c**) The presence of R248Q after DCA treatment causes a reduction of autophagy flux. HL60 cells were transfected with plasmids expressing wt p53 or mt p53. After 24 h transfection, cells were incubated with 10 mM DCA for 24 h. Autophagy flux was monitored by the addition of 50 μM CQ for 4 h before collecting the cells for western blot analysis. Graphs represent the quantification of the western blots of 3 experiments.

To further investigate whether DCA inhibits macroautophagy via mt p53, we silenced or overexpressed R248Q in the previously portrayed AML cell lines (Fig. 7). First, we analyzed in NB4 cells the effect of DCA on the mRNA levels of different autophagy genes after reduction of R248Q by siRNA (Fig. 7a). R248Q down-regulation efficiently enhanced the expression of the autophagy genes indicating that the presence of R248Q inhibited the macroautophagy pathway. The knockdown of R248Q partially blocked the inhibitory effect of DCA. In particular, the inhibition of *BCEN1* mRNA by DCA was entirely prevented after reduction of R248Q. Beclin-1, the protein encoded by *BCEN1* gene, is a crucial component of nucleation and maturation of macroautophagy pathway, one of the early steps of macroautophagy. ATG5/ATG12 and LC3 mediate the elongation of the phagophore^43^. Based on this data, R248Q could have an inhibitory effect on the formation of the autophagosome at the early steps of the macroautophagy pathway. In addition, DCA did not increase *mt p53* mRNA in NB4 cells treated with control siRNA (Fig. 7a). This shows that DCA effects mainly rely in protein stability as previously proposed^29^.

Hence, we next checked the autophagy flux after R248Q down-regulation (Fig. 7b). As we previously observed, DCA reduced autophagy flux in control siRNA transfected cells. Interestingly, the flux was restored after p53 knockdown. Furthermore, ectopic expression of wt p53 and R248Q in p53-null HL60 cells caused a reduction of the autophagy flux in untreated cells (Fig. 7c). In the presence of DCA, only R248Q further decreased the autophagy flux. All these results demonstrate the repressive role of mutant p53-R248Q in macroautophagy and suggest that the DCA-induced inhibition of macroautophagy could be due to the increase in mutant p53-R248Q levels.

### Co-treatment of DCA and 17-AAG caused inhibition of macroautophagy in p53 dependent manner

**Figure 8:**
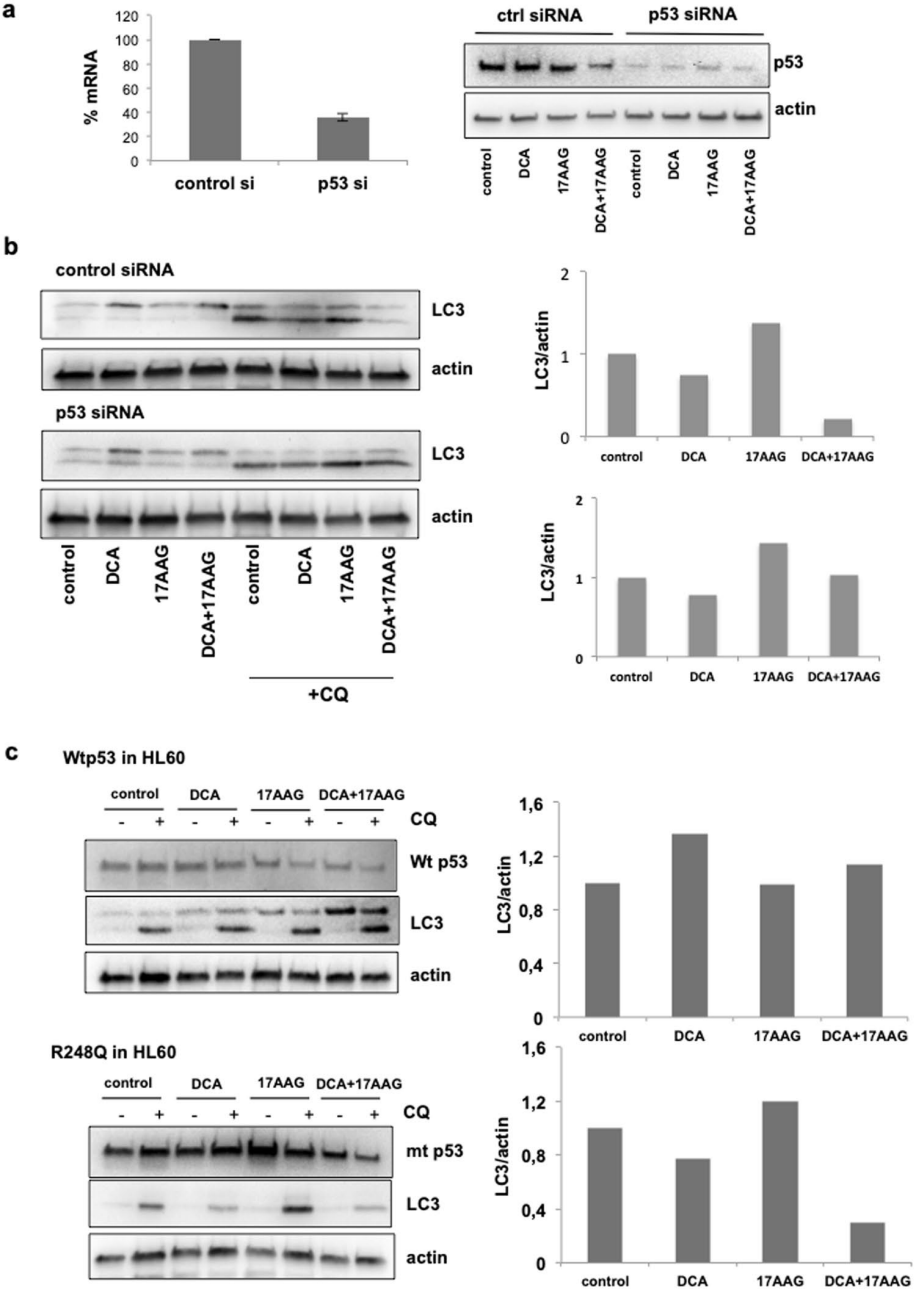
The combination DCA + 17-AAG causes inhibition of macroautophagy in the presence of R248Q. (**a**) NB4 cells were transfected with control siRNA and p53 siRNA. Knockdown efficiency was validated by qPCR and western blot. (**b**) DCA + 17-AAG co-treatment causes inhibition of autophagy flux in presence of R248Q. After 72 h transfection with the indicated siRNAs, NB4 cells were treated with 10 mM DCA and/or 1 µM 17-AAG. Autophagy flux was monitored by detection of LC3-II under CQ treatment. (**c**) R248Q inhibits autophagy flux stimulated by metabolism stress. HL60 cells were transfected with plasmids encoding wt p53 or R248Q and after 24 h, DCA and/or 17-AAG were added for 24 h. CQ was used to monitor autophagy flux. Graphs represent the quantification of the western blots.

To investigate the effect of wt p53 or R248Q on macroautophagy after treatment with DCA and/or 17-AAG, we carried out experiments to overexpress and knockdown both genes. The efficiency of the knockdown of R248Q was confirmed by qPCR and western blot analysis in NB4 cells (Fig. 8a). Whereas in cells transfected with siRNA control DCA + 17-AAG decreased the autophagy flux (Fig. 8b, top panel), reduction of R248Q by siRNA re-established the autophagy flux (Fig. 8b, bottom panel). This data demonstrate a critical role of R248Q in inhibiting autophagy. To further explore the role of R248Q in autophagy, HL60 cells were transfected with plasmids encoding wt p53 or R248Q. Autophagy flux analysis showed that when R248Q was overexpressed, autophagy flux decreased after DCA + 17-AAG co-treatment (Fig. 8c, bottom panel). However, overexpression of wt p53 had little or no effect on autophagy flux (Fig. 8c, top panel). These data are consistent with the idea that this mt p53 has an inhibitory effect on the autophagy flux stimulated by metabolism stress.

## Discussion

Cells growing in low resources or under metabolic stress use autophagy to survive. We show here that metabolic stress regulates different forms of autophagy depending on p53 status. DCA induces metabolic stress and p53 accumulation. In cells expressing wt p53 or lacking p53 expression, DCA does not affect the autophagy flux. In cells expressing the p53 mutation R248Q, metabolic stress represses macroautophagy and promotes p53-R248Q accumulation. In a feed back loop, R248Q represses further macroautophagy inducing even higher accumulation of R248Q protein levels. We show that 17-AAG short-circuits this loop by inhibiting Hsp90, which releases R248Q bound to Hsc70. In this condition, Hsc70 induces massive R248Q degradation by CMA. Interestingly, wt p53 is not affected by this treatment because is not mainly stabilized by Hsp90. Therefore; the DCA + 17-AAG is effective to eliminate mt p53.

It is now established that mt p53 acquires oncogenic functions to drive cell migration, invasion, and metastasis and p53 mutation does not represent the equivalent of p53 loss^7^. Furthermore, not all p53 mutants have the same GOF activities. Different p53 mutations impart unique activities to stimulate the development of various tumor types. Therefore, it is essential to study each p53 mutant independently to devise different target and therapeutic strategies. It has been reported that mt p53 could be targeted for proteasomal or autophagy degradation^24,25^, however remain unclear how mutant R248Q is degraded. In the present study, we investigated different strategies to promote the elimination of R248Q in different cellular conditions. We specifically focus on p53-R248Q mutant because is one of the most frequently found in a wide range of human cancers, especially in AML. It would be interesting to investigate if other p53 mutants are degraded by the same mechanisms than p53-R248Q. Although, it is possible that different degradation mechanisms will be activated depending on the physiological conditions, e.x. metabolic stress, and depending on the mutation. Specific mt p53 proteins accumulate to high levels in tumor cells due to defects in their degradation. Additionally, multiple stress signals can induce their stabilization and promote its GOF. This favors the development of more aggressive tumors^11^. mt p53 stabilization could be due to interaction with Hsp70 and Hsp90 chaperones that protect mt p53 from degradation^23^.

We found that autophagy and not proteasomal degradation is the primary pathway responsible for the effective elimination of R248Q. The use of DCA and 17-AAG allowed us to determine the alternative types of the autophagy pathway engaged to degrade R248Q under different conditions. DCA stabilizes R248Q by enhancing its binding with the Hsp90 chaperone and inhibiting the macroautophagy pathway. In contrast, inhibition of Hsp90 function by 17-AAG induces macroautophagy and promotes R248Q degradation. When macroautophagy is repressed under confluent conditions, CMA degrades mt p53^26^ CMA contributes to energetic cellular balance and it is activated by metabolic stress^44^. We show here that DCA-induced PDK1 inhibition constrains macroautophagy and by inducing metabolic stress activates CMA. This explains why DCA + 17-AAG causes higher R248Q destabilization than 17-AAG alone.

The Hsc70 cytosolic chaperone is essential in mediating CMA^45^. It is also part of Hsp90 complex, and it can be found associated with different mt p53 proteins^26,46^. We propose that DCA pre-treatment increases the interaction of R248Q with the chaperone complex, including Hsc70, leading to its stabilization and inhibition of macroautophagy. The subsequent addition of 17-AAG caused the release of Hsp90 from the complex but without affecting the interaction with Hsc70. This promotes CMA-mediated R248Q degradation. Similar results were found in a study using an oxazoline analog of apratoxin A (oz-apraA)^47^. By inhibiting Hsp90 function, oz-apraA increases the interaction of Hsp90 clients to Hsc70/Hsp70 chaperones and promotes their degradation by CMA.

The crosstalk between different forms of autophagy pathways has been reported by various studies and the Hsc70 chaperone has been proposed as a candidate for acting as a cross-talking molecule between macroautophagy and CMA^47^. Cells with impaired CMA function were able to promote protein degradation through up-regulation of macroautophagy^48^ and vice versa, inhibition of macroautophagy also contributes to further induction of CMA^45^. Macroautophagy and CMA communicate with each other and Hsc70 may be a fundamental element for this crosstalk^49^.

In summary, we provide different strategies to eliminate mutant R248Q p53 using 17-AAG. Our data show that 17-AAG induces macroautophagy to eliminate R248Q under normal growth conditions. Energy stress stimulates the accumulation of R248Q with molecular chaperones and enhances its inhibitory effect on macroautophagy. Under this stress condition, 17-AAG still can remove R248Q protein through CMA pathway. Understanding the mechanism of mt p53 degradation may help in the development of new therapeutic approaches, and it will be useful for the treatment of patients carrying p53 mutations.

## Materials and Methods

### Cell cultures

AML (OCI-AML-3, NB4 and HL60) cell lines were cultured as previously described^29^. OCI-AML-3 cells harbor wt p53, NB4 carries mt p53 (R248Q) and HL60 is a p53^−/−^ cell line. Cells were grown at 37 °C and 5% CO_2_ in a humidified atmosphere.

### Reagents, siRNAs, plasmids and transfection

DCA was purchased from Santa Cruz. 17-AAG was from Selleck. MG132 was purchased from Calbiochem. Cycloheximide, wortmannin, bafilomycin A, chloroquine and 3MA were purchased from Sigma-Aldrich. Wt and mutant p53 constructs were a gift from Dr. Shannon C Kenney. Transfection of wt p53 and R248Q was carried out using Lipofectamine RNAiMAX (Invitrogen) in Opti-MEM (Invitrogen), according to the manufacturer’s instructions. p53 siRNA was a gift from Dr Xirodimas and it was ON-TARGETplus SMARTpools (mixture of 4siRNA) from Dharmacon. Two siRNA duplexes were used to knockdown Hsc70 as previously described^50^. siRNAs were synthesized by Eurofins MWG Operon. Cells (2 × 10^6^ in 100 μl) were transfected with 100 nM Hsc70 siRNA, p53 siRNA or control siRNA by electroporation using Amaxa SF Cell line 4D-Nucleofector kit (Lonza Bioscience). Cells were harvested 24 to 72 h post-transfection.

### Cell proliferation, viability

Cell viability and cell numbers were determined using the Muse® Cell Analyzer (Millipore) as previously described^29^.

### Western blot analysis

Primary antibodies against HSP90 (C45G5) and β-actin were purchased from Cell Signaling Technology. LC3B antibody was from GenTex. HSC70 (13D3) was purchased from Abcam. The anti-p53 antibody (DO-1) was a gift from Dr Xirodimas. Cell extracts were lysed in 2x SDS sample buffer. Proteins were resolved by SDS-PAGE and transferred to nitrocellulose or PVDF membranes using the Trans-Blot® Turbo™ Transfer System **(**Bio-Rad). Peroxidase-coupled anti-mouse and anti-rabbit secondary antibodies were used at a dilution of 1:10.000 (Sigma). Bound antibodies were detected by enhanced chemiluminescence (Millipore). To obtain the image for the western blotting the Molecular Imager Gel Doc XRS system (Biorad) was used, this system provides a reliable and sensitive imaging of chemilunescence western blots. For the quantification and analysis, we used the Image Lab Software (Biorad), a powerful and specific software for acquisition, analysis and quantification of blot images.

### Measure of p53 half-life

To measure the half-life of mt p53, NB4 cell lines were treated with 10 mM DCA and/or 1 µM 17-AAG. After 24 hours, the protein synthesis inhibitor cycloheximide (20 µg/ml), was added at different time points. Cells extracts were prepared and the remaining mt p53 protein levels determined by Western blotting. For the quantification and analysis of the protein levels, we used the Image Lab Software (Biorad).

### Immunoprecipitation

Cells were lysed in NP-40 buffer: 0.5% NP-40, 50 mM HEPES pH 7.5, 100 mM NaCl, 5 mM EDTA, 1 mM DTT and complete protease inhibitor mixture (Roche). Lysates were used for co-immunoprecipitation using 1 μg of antibodies: anti-p53 (DO-1) or anti-HSC70 as indicated. Dynabeads protein G (Thermo Fisher) was used following manufacturer’s instructions.

### RT-PCR and DNA sequencing

Total RNA was extracted using NucleoSpin RNA isolation columns (Macherey-Nagel), reverse transcription was carried out using random primers. Quantitative PCR was performed as described previously^51^ with SsoADV SYBR Green qPCR SuperMix (Biorad) and a CFX Connect^TM^ Real-Time qPCR machine (Biorad). p53 and actin primers were previously described^29^. *Beclin 1*, *ATG5*, *LC3a and LC3b*^52^ and *ATG12*^28^ primers were previously described. All samples were normalized to *β-actin* mRNA levels.

### Quantification of autophagy flux

Flux was monitored by the addition of CQ (50 µM) for 4 h at the end of the incubation periods. Flux was determined by subtracting control sample from CQ treated samples, thus reflecting the amount of LC3-II that accumulated in the 4 h following CQ addition.

### Statistical analysis

Statistical analysis was performed using the Student’s *t* test: *p < 0.05; **p < 0.01; ***p < 0.001. Values were expressed as the mean ± the standard error of the mean (SEM).

## Supplementary information

Supplementary Figure 1
