# Supplementary material for: Metabolic stress controls mutant p53 R248Q stability in acute myeloid leukemia cells
Source: Sci Rep. 2019 Apr 4;9:5637. doi: 10.1038/s41598-019-42220-y (PMC6449403; doi:10.1038/s41598-019-42220-y)
Supplement: Supplementary file 1 — Supplementary Figure 1 [file 41598_2019_42220_MOESM1_ESM.pdf]

Metabolic stress controls mutant p53 R248Q stability in acute myeloid leukemia cells

Nerea Allende-Vega, and Martin Villalba

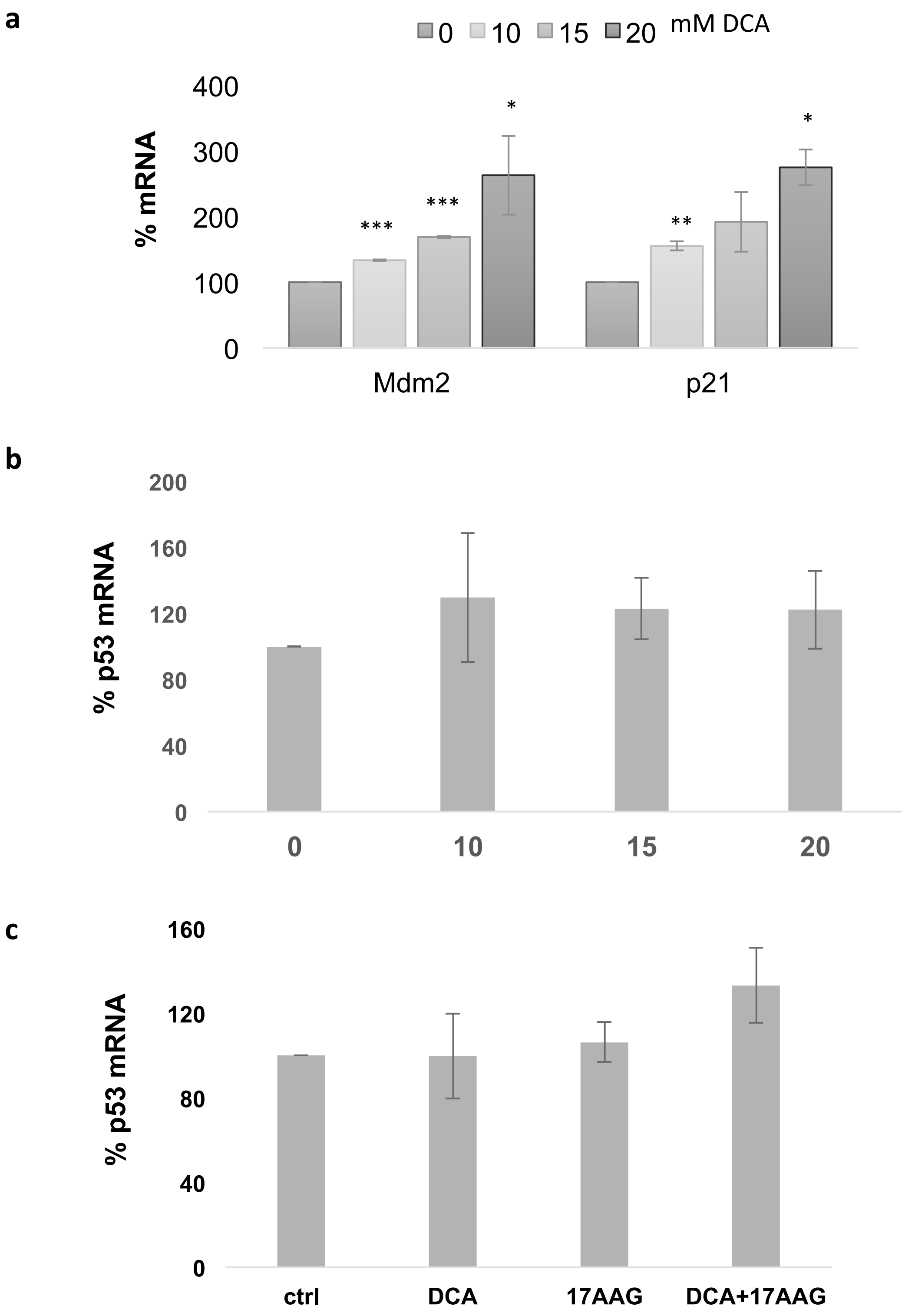

Supplementary Figure 1. (a) DCA induces wt p53 transcription activation in OCI-AML-3 cell lines. DCA increases MdM2 and p21 mRNA levels after 24h of treatment with different concentration of DCA. (**b and c).** The mt p53 **(**R248Q) mRNA levels in NB4 cell line were nor affected after DCA treatment and co-treatment of DCA and 17AAG**.**
